# The Clinical Efficacy of Different Relaxation Exercises on Intraocular Pressure Reduction: A Meta-Analysis

**DOI:** 10.3390/jcm13092591

**Published:** 2024-04-28

**Authors:** Bing-Qi Wu, Hou-Ting Kuo, Alan Y. Hsu, Chun-Ju Lin, Chun-Ting Lai, Yi-Yu Tsai

**Affiliations:** 1 Department of General Medicine, China Medical University Hospital, Taichung 404, Taiwan; jacky870914@gmail.com (B.-Q.W.); a0934305326@gmail.com (H.-T.K.); 2 Department of Ophthalmology, China Medical University Hospital, China Medical University, Taichung 404, Taiwan; alanhsu1221@gmail.com (A.Y.H.); yiyutsai@seed.net.tw (Y.-Y.T.); 3 School of Medicine, College of Medicine, China Medical University, Taichung 404, Taiwan; 4 Department of Optometry, Asia University, Taichung 413, Taiwan

**Keywords:** mindfulness-based stress reduction, yoga, relaxation, complementary therapy, glaucoma, ocular hypertension

## Abstract

**Objective**: The aim of this study was to synthesize the available evidence on the clinical efficacy of different relaxation exercises on intraocular pressure (IOP) reduction. **Methods**: A systemic search of PubMed, Embase, Cochrane CENTRAL, and Web of Science was undertaken from the earliest record to 10 April 2024. Peer-reviewed studies that reported on healthy individuals and glaucoma patients engaging in relaxation exercises for at least three weeks were included. The primary outcome was changes in IOP levels from baseline, before the commencement of relaxation exercises, to post-exercise. Our statistical analysis employed a random-effects model, with effect sizes reported using Hedges’ g. **Results**: Twelve studies were included, totaling 764 eyes (mean participant age ranging from 21.07 to 69.50 years). Relaxation exercises significantly reduced IOP, with Hedges’ g being −1.276 (95% CI: −1.674 to −0.879) and I^2^ = 84.4%. Separate subgroup analyses showed that breathing exercises (Hedges’ g = −0.860, *p* < 0.0001), mindfulness-based stress reduction (MBSR) (Hedges’ g = −1.79, *p* < 0.0001), and ocular exercises (Hedges’ g = −0.974, *p* < 0.0001) were associated with reduced IOP levels. The reduction in IOP following the relaxation exercises was found to be associated with baseline IOP either greater than (Hedges’ g = −1.473, *p* < 0.0001) or less than 21 mmHg (Hedges’ g = −1.22, *p* < 0.0001). Furthermore, this effect persisted with follow-up durations of less than (Hedges’ g = −1.161, *p* < 0.0001) and more than one month (Hedges’ g = −1.324, *p* < 0.0001). **Conclusions**: The current meta-analysis indicates that relaxation exercises can significantly reduce IOP levels. Relaxation exercises are a potential class of novel treatments for glaucoma patients that deserve further evaluation.

## 1. Introduction

Glaucoma is a progressive optic neuropathy characterized by the loss of retinal ganglion cells and is a significant global health problem. Recent estimates have shown that the number of people affected by primary open-angle glaucoma (POAG) and primary angle-closure glaucoma (PACG) globally was approximately 60.5 million [1,2]. The global prevalence rates for primary open-angle glaucoma (POAG) and primary angle-closure glaucoma (PACG) vary across different regions. For instance, one study reported that the prevalence of primary open-angle glaucoma (POAG) was highest in Africa, estimated at 4.20%. In contrast, the prevalence of primary angle-closure glaucoma (PACG) was seen to be highest in Asia, estimated at 1.09% [2].

Several studies have highlighted the association between glaucoma and medical conditions like hypertension and dyslipidemia [3]. While less studied in comparison, emerging research has also drawn links between glaucoma and mental health disorders such as depression. Current guidelines primarily focus on addressing medical comorbidities in glaucoma patients while overlooking potential psychiatric comorbidities [3]. Studies have reported that 18% to 36% of individuals with glaucoma also experience concurrent mental disorders such as depression and anxiety [4]. This prevalence of mental health-related comorbidities among glaucoma patients points towards the potential therapeutic efficacy of mental health interventions for this patient group. Such therapeutic applications of mental health interventions for glaucoma patients could be promising given the bidirectional links discovered between glaucoma and certain psychological conditions. For example, previous studies have highlighted synaptic loss and altered connectivity in neural networks in individuals with mental health issues like depression and anxiety. These changes in the central neural population may also impact retinal ganglion cells, potentially exacerbating the clinical outcomes of glaucoma patients with concurrent mental health issues [5]. Those with mental health issues have also been found to have elevated levels of stress hormones like cortisol due to imbalance between the parasympathetic and the sympathetic systems. These stress hormones can reduce ocular blood flow through their effect on the autonomic nervous systems, worsening glaucomatous neuropathy [6,7]. Conversely, glaucoma can also affect patients’ mental health due to diminished vision-related quality of life [8]. Therefore, based on these potential links between mental health and glaucoma (refer to Supplementary Figure S1), various relaxation techniques rooted in the principles of mindfulness have been developed and studied in glaucoma patients. Mindfulness involves directing attention to the present moment, serving as a guiding principle for practices like meditation, yoga, and other relaxation techniques, such as breathing exercises. Studies have described these techniques to positively affect neural structures, autonomic nervous system hormonal pathways, and ocular blood flow through increased cerebral blood flow, improved neural structure and activation, increased grey matter volume, and decreased stress hormones [9,10]. However, despite these promising findings from the literature, studies on the efficacy of mental health interventions for glaucoma patients have been limited to single studies with small patient numbers. Comprehensive data on the effects of different relaxation and mindfulness-based therapies (e.g., yoga, mindfulness-based stress intervention, and breathing exercises) on intraocular pressure (IOP) in a single study are lacking. Furthermore, there have been contradictory reports in the literature, with one study showing that certain exercises resulted in increased intraocular pressure [11]. These conflicting findings highlight the necessity for a more comprehensive understanding of alternative treatments for glaucoma. Annual direct medical costs related to glaucoma have been estimated at USD 2.9 billion, emphasizing the urgent need for effective interventions. This urgency is further highlighted by the fact that these costs are often underestimated when surgical interventions related to glaucoma are not accounted for. One study demonstrated that the total direct costs for glaucoma patients are 49.5% higher when glaucoma patients receive operative procedures [12]. Although there is a lack of studies investigating the cost-effective potential of mindfulness-based relaxation interventions among glaucoma patients, if such relaxation interventions can be proven to reduce intraocular pressure (IOP) and reduce the need for surgical interventions, this could have significant cost-saving potential for this important clinical condition. This study aimed to fill these knowledge gaps by conducting a meta-analysis to evaluate the impact of specific mindfulness-based relaxation exercises on changes in IOP and their overall efficacy among patients with glaucoma or ocular hypertension.

## 2. Materials and Methods

### 2.1. Search Strategy and Study Eligibility

This systemic review and meta-analysis was guided by the Preferred Reporting Items for Systematic Reviews and Meta-Analyses (PRISMA) [13]. The protocol was preregistered on PROSPERO (CRD42024520372).

Regarding inclusion criteria, eligible studies incorporating mindfulness-based relaxation interventions, including visualization, deep breathing, and yoga, were included. These interventions were evaluated for their effects on individuals diagnosed with glaucoma or ocular hypertension (OHT) or among participants without these conditions (i.e., healthy participants). Additionally, this meta-analysis only recruited studies that quantitatively evaluated the effects of such interventions through IOP measurements (refer to Supplementary Figure S2). In terms of the specific search strategy, two authors conducted independent electronic searches in the databases mentioned above using the following combination of terms (“mindfulness” OR “relaxation techniques” OR “yoga” OR “meditation” OR “deep breathing” OR “visualization” OR “self-massage”) AND (“intraocular pressure” OR “glaucoma” OR “ocular hypertension” OR “normal tension glaucoma”). An extensive electronic search was conducted in online databases, including PubMed, Embase, Cochrane CENTRAL, and Web of Science, from the earliest record until 10 April 2024. As part of this search, two authors (BQW and HTK) initially screened the identified titles and abstracts for eligibility. The reference lists of an identified review article were also checked. The authors also performed additional manual searches. When a consensus was not achieved between the two authors, consultation with a third study author was sought. The study imposed no language restrictions and did not specify a pre-set study type.

Regarding exclusion criteria, this meta-analysis excluded the following: (1) animal studies, (2) studies for which full texts could not be obtained or no data were available, (3) studies lacking outcome measures that included IOP, (4) studies without pre- and post-intervention assessments or lacking measurements between pre- and post-intervention, (5) studies lacking a placebo-controlled group, (6) studies with patients who did not perform relaxation techniques for a duration of at least 3 weeks. Informed consent and the need for institutional review board approval were waived by the China Medical University Hospital Institutional Review Board due to the lack of patient-identifying data.

### 2.2. Appraisal of Methodological Quality

To evaluate the methodological quality of the included studies, the authors adopted the Cochrane risk of bias tool for randomized studies (version 2, RoB 2, London, UK). This appraisal tool consists of six main items for evaluating study quality: the randomization process, intervention adherence, missing outcome data, outcome measurement, selective reporting, and the overall risk of bias [14].

In the intervention adherence domain of RoB 2, the authors chose the per-protocol evaluation among two options presented for literature assessment: intention-to-treat evaluation (intervention assignment) and per-protocol evaluation (intervention adherence) [14].

### 2.3. Data Extraction and Management

Two independent authors extracted data from the evaluated studies. The extracted data included first author, publication year, country, study design, patient age, baseline IOP, duration of follow-up, and relaxation regimens (type and duration). Data extraction, conversion, and merging results from various study arms were implemented. These processes strictly adhered to the recommendations outlined in the Cochrane Handbook for Systematic Reviews of Interventions and were consistent with the literature [15].

### 2.4. Statistical Analyses

This meta-analysis was conducted with a random-effects model [16] using the Comprehensive Meta-Analysis software (version 3, Biostat, Englewood, NJ, USA). A two-tailed *p*-value less than 0.05 was defined as statistically significant.

Hedges’ g and 95% confidence intervals (CIs) were utilized to estimate the efficacy of relaxation techniques (i.e., changes in IOP). Hedges’ g values of 0.2, 0.5, and 0.8 were classified as thresholds to represent small, moderate, and large effect sizes, respectively [17]. The associated calculation involved the changes in IOP before and after the relaxation techniques. A negative effect size value signifies a favorable outcome for relaxation techniques relative to the baseline. Heterogeneity was quantified using I^2^ and Cochran’s Q tests. I^2^ values of 25%, 50%, and 75% were classified as low, moderate, and high heterogeneity, respectively [18].

Subgroup analyses, such as initial presenting IOP and follow-up duration, were also conducted to assess the impact that type of mindfulness-based relaxation intervention has on heterogeneity.

One-study removal methods were also used to determine whether there was a statistically significant change in the summary effect size after removing a particular trial from the analysis [19].

Guidelines from the Cochrane Handbook for Systematic Reviews of Interventions were used to evaluate for potential publication bias [20]. Funnel plots were generated and visually inspected for symmetry. Egger’s regression tests were conducted when 10 or more datasets were available.

## 3. Results

### 3.1. Study Characteristics

A total of 3352 studies were initially identified. Following screening, 30 studies were selected for full-text assessment. Among the 17 excluded studies, three did not contain a sufficient duration of follow-up period. Fourteen studies were excluded because their trial designs did not include a placebo group. One study was excluded as its research participants were identical to those of another study [21]. Ultimately, twelve studies (consisting of 764 eyes) were incorporated into our research [21,22,23,24,25,26,27,28,29,30,31,32,33] (refer to Supplementary Figure S3 and Table 1). The included studies were from Asia (n = 9), the Middle East (n = 2), and Europe (n = 2). In summary, a total study population of 395 patients (764 eyes) were included. The mean age of participants ranged from 21.07 years to 69.50 years. The female participants ranged from 20% to 78% across all studies.

### 3.2. Quality Assessment and Risk of Bias

Most of our recruited studies had a low risk of bias (Supplementary Figure S4). Three of the studies [26,28,32] were categorized under “some concerns” because of either a lack of details on allocation concealment or due to the potential occurrence of unbalanced non-protocol interventions. Two studies [21,31] were categorized under “high risk” of bias due to multiple domains within these studies being assessed as having “some concerns”. These studies [21,31] showed some bias concerns in two domains, randomization and intervention performance, respectively.

### 3.3. Outcome of Intraocular Pressure

**Figure 1:**
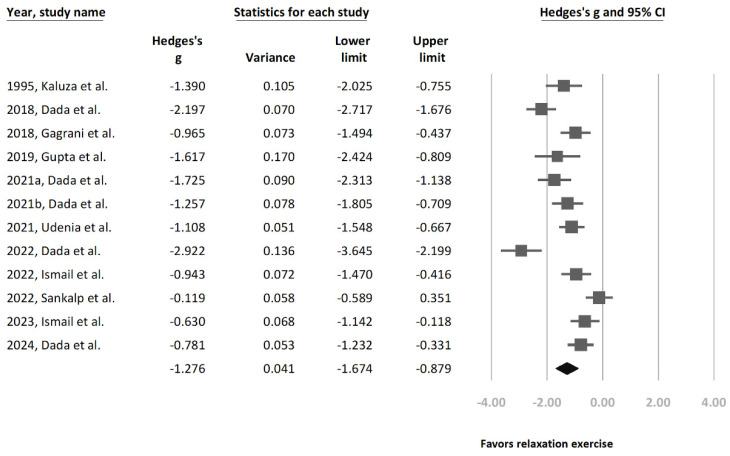
Forest plot presenting Hedges’ g in terms of overall intraocular pressure (IOP) changes before and after relaxation exercise [21,22,23,24,25,26,27,28,29,30,32,33].

**Figure 2:**
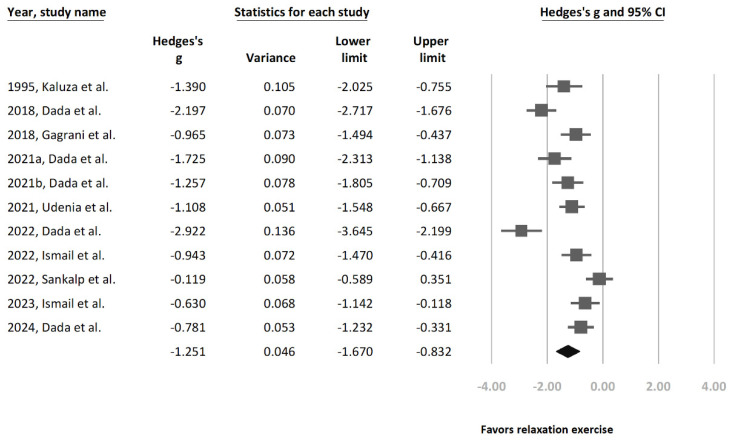
Forest plot presenting Hedges’ g in terms of overall intraocular pressure (IOP) changes in glaucoma and ocular hypertension patients before and after relaxation exercise [21,22,23,24,25,26,27,29,30,32,33].

Overall, this study found mindfulness-based relaxation interventions from our 12 recruited studies to have a statistically significant effect in reducing IOP (Hedges’ g = −1.276 [95% CI = −1.674 to −0.879], *p* < 0.0001, I^2^ = 84.4%) (Figure 1). To further ascertain the efficacy of relaxation exercises among patients with glaucoma or OHT, this study conducted a separate analysis that excluded studies that contained only healthy participants [28]. After exclusion, this separate subgroup analysis of the remaining 11 studies (Figure 2) still showed a significant statistical relationship between relaxation exercises and IOP reduction (Hedges’ g = −1.251 [95% CI = −1.67 to −0.832], *p* < 0.0001, I^2^ = 85.5%).

**Figure 3:**
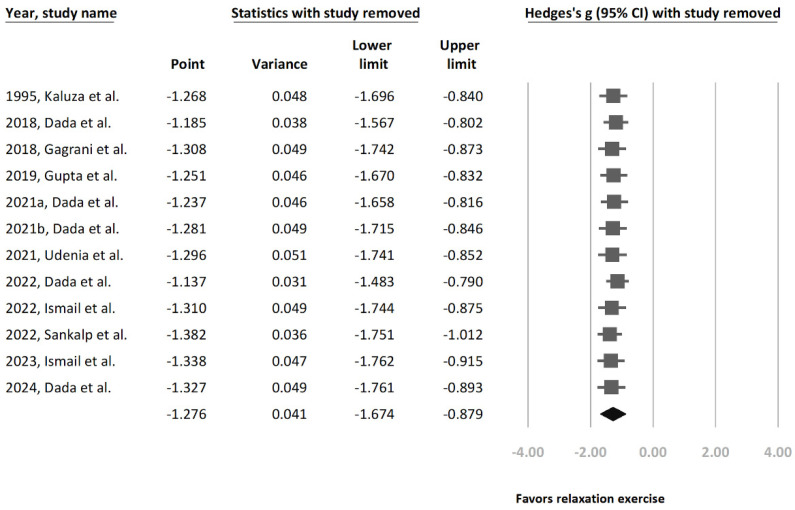
Sensitivity analysis using the one-study removal method. The main result did not change significantly after removing any one of the included studies. All analyses showed statistically significant effects of relaxation exercise on IOP reduction [21,22,23,24,25,26,27,28,29,30,32,33].

A high heterogeneity was observed in both subgroup analyses (Figure 1 and Figure 2). Therefore, an additional sensitivity analysis was conducted using the one-study removal method. This analysis showed a consistently statistically significant effect on IOP reduction in terms of relaxation exercises among the recruited study population (Hedges’ g = −1.276 [95% CI: −1.674 to −0.879]) (Figure 3).

**Figure 4:**
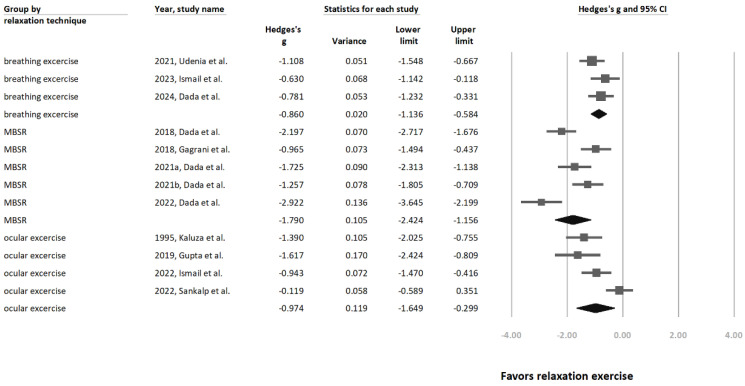
Forest plot presenting subgroup analysis based on different techniques of relaxation exercise [21,22,23,24,25,26,27,28,29,30,32,33].

A subgroup analysis based on relaxation technique categories (Figure 4) was also conducted. As part of this subgroup analysis, the recruited studies were organized based on the categories of mindfulness-based relaxation interventions used. The categories of mindfulness-based relaxation interventions are mindfulness-based stress reduction (MBSR), breathing exercises, and ocular exercises. Among the breathing exercise subgroup, a significant relationship was observed between breathing-related mindfulness-based relaxation interventions and IOP reduction (Hedges’ g = −0.860 [95% CI: −1.136 to −0.584], *p* < 0.0001, I^2^ = 5.1%). In the MBSR group, a significant relationship was observed between MBSR-related intervention and IOP reduction (Hedges’ g = −1.79 [95% CI = −2.424 to −1.156], *p* < 0.0001, I^2^ = 83.6%). In the ocular exercise group, a significant relationship was observed between ocular exercise-related intervention and IOP reduction (Hedges’ g = −0.974 [ 95% CI = −1.649 to −0.299], *p* < 0.0001, I^2^ = 80.7%).

In the predefined subgroup analyses focusing on initial presenting IOP (Supplementary Figure S5), the baseline IOP was classified into two categories: those with elevated initial IOP (defined as greater than 21 mmHg) and those with low initial IOP (less than 21 mmHg). Among studies with elevated initial IOP, a significant reduction in IOP was observed at the final follow-up after mindfulness-based relaxation interventions (Hedges’ g = −1.473 [95% CI = −2.711 to −0.234], *p* < 0.0001, I^2^ = 92.7%). Among studies with a low initial IOP, a significant reduction in IOP was observed at the final follow-up after mindfulness-based relaxation interventions (Hedges’ g = −1.22 [95% CI = −1.633 to −0.806], *p* < 0.0001, I^2^ = 81.3%).

As part of our subgroup analyses focusing on follow-up time duration (Supplementary Figure S6), this meta-analysis categorized studies into (1) studies with a follow-up time of one month and over and (2) those with a follow-up period of less than one month. Within the subgroup analysis that recruited studies that contained a follow-up of one month and over, a significant relationship was seen between the mindfulness-based relaxation interventions employed and the reduction in IOP at final follow-up (Hedges’ g = −1.324 [95% CI = −1.723 to −0.924], *p* < 0.0001, I^2^ = 75.3%). Within the subgroup analysis that recruited studies that contained a follow-up of less than one month, a significant relationship was seen between the mindfulness-based relaxation interventions employed and the reduction in IOP at final follow-up (Hedges’ g = −1.161 [95% CI = −2.126 to −0.196], *p* < 0.0001, I^2^ = 92.7%).

For publication bias assessment, a funnel plot (Supplementary Figure S7) of the 12 included studies displayed some asymmetry in effect size (Hedges’ g) distributions. Egger’s regression test showed a *p*-value of 0.037, indicating the existence of potential publication bias.

## 4. Discussion

### 4.1. Novel Findings

Our meta-analysis of 12 related studies revealed a significant overall reduction in IOP following relaxation and mindfulness-based intervention. Subgroup analysis based on the different categories of mindfulness-based relaxation interventions, baseline IOP (less than and greater than 21 mmHg), and follow-up duration (more than or less than one month) all further demonstrated a significant relationship between mindfulness-based relaxation interventions and the reduction in IOP at final follow-up among such patients.

### 4.2. Clinical Implications

Current guidelines on glaucoma treatments lack recommendations for alternative health approaches [34]. This is partly due to the scarcity of studies systemically evaluating the effects of such novel approaches. Our findings, therefore, have real-world implications for glaucoma patients.

### 4.3. Comparisons to Other Studies

Our results are partly in line with the literature. For example, a meta-analysis of five studies by Zaher et al. [11] reported a significant reduction in IOP following mindfulness-based relaxation interventions among glaucoma patients (standardized mean difference (SMD) = −2.02; range: −3.16 to −0.89). Zaher et al. also conducted a subgroup analysis based on the interventions used, revealing that mindfulness sessions resulted in a significant percentage of IOP reduction (31.8%), and ocular exercises showed an overall IOP reduction of 22.0%. However, such effects were not unanimous across all interventions assessed. For example, inverted yoga postures and rigorous physical exercise led to a paradoxical increase in IOP. Zaher et al. also reported that the total duration of the interventions showed no correlation with the percentage of IOP reduction. While these results partially complemented our own, some minor differences exist. Firstly, Zaher et al. used the IOP reduction percentage to assess outcomes among the studies recruited, while we used Hedges’ g (effect size) to estimate effects. Another issue concerns the lack of standardization of the mindfulness-based relaxation interventions employed. For example, Zaher et al. and many of the studies from our meta-analysis lacked detailed descriptions concerning the mindfulness-based relaxation interventions. Any modification of interventions could potentially influence their effects on IOP and contribute to heterogeneity among studies. Zaher et al. hinted at such effects by demonstrating that certain subtypes of yoga exercises, like head-down positions, may increase IOP.

This study also assessed the effect of different forms of yoga on IOP by categorizing the yoga exercises into breathing and intra-ocular exercises. The ocular exercise subgroup exhibited a high effect size corresponding to the quantitative results of previous meta-analyses (refer to Figure 4). Comparatively, Chetry et al. [35] also showed that ocular exercise reduced IOP with a Hedges’ g of −0.94. However, high heterogeneity was observed among the subgroup of studies concerning ocular exercises. This possibly hints at the variability in the ocular exercises employed and in the included studies’ baseline characteristics (such as sample sizes and baseline IOPs).

Regarding the breathing exercise subgroup analysis (refer to Figure 4), the effect size reached −0.86, indicating a relatively large impact on IOP reduction. This finding is consistent with several cohort studies [36,37,38,39,40].

Regarding our baseline IOP subgroup analysis (refer to Supplementary Figure S5), our results showed that a significant reduction in IOP was observed in both subgroups (high baseline IOP and low baseline IOP) at the final follow-up after mindfulness-based relaxation interventions. Such effects from baseline IOP have been hinted at by studies which showed higher baseline IOP to affect glaucoma progression after standard pharmacological and surgical treatments [41].

Regarding impact based on follow-up durations, the subgroup analysis results showed a significant association with IOP reduction after interventions among studies with a follow-up duration of more than one month and those with follow-up intervals of less than one month. Our study also showed moderate to high heterogeneity among the studies assessed. This partly aligns with studies like Udenia et al.’s [33], where mindfulness-based relaxation interventions were significantly associated with IOP reduction when received over one-month, three-month, and six-month durations. However, the literature is not unanimous, as Zaher et al. reported in their statistical analysis that no association existed between IOP reduction and duration of mindfulness-based relaxation interventions [11]. Future studies are needed to confirm our findings further.

### 4.4. Strengths and Limitations

One of the strengths of our study is that it is the first to systemically evaluate the clinical efficacy of mindfulness-based relaxation interventions among glaucoma patients in a single meta-analysis. However, there are also limitations. Firstly, there was variability in the regimens of the relaxation exercises employed (e.g., frequency and duration) and a lack of comprehensive descriptions concerning the exercises assessed. This is partly due to the current lack of standardization for yoga, meditation, and other mindfulness-based relaxation interventions in the literature [42]. Therefore, as a consequence, this meta-analysis cannot provide exact descriptions of the mindfulness-based relaxation interventions that met our inclusion criteria. Other related meta-analyses lacked procedural descriptions of these relaxation interventions as part of their inclusion criteria [42,43]. This may have an impact on the generalizability of our findings. Another limitation was that we did not evaluate the cost-effectiveness of mindfulness-based exercises for glaucoma patients. These considerations are crucial for patients, as they can impact treatment adherence and ultimately affect patient outcomes. Theoretically, if clinical efficacy is further confirmed in future studies regarding mindfulness-based interventions for glaucoma patients, it could be inferred that the reduction in intraocular pressure associated with these interventions might lead to decreased surgical interventions for glaucoma patients, resulting in indirect cost savings. However, future studies are needed to confirm such effects on costs. Another limitation is the low number of studies recruited, which limited the power of our meta-analysis. Other confounders include the unknown baseline ocular structural and vascular functions among the recruited patients. For example, studies have hinted at a relationship between thinner retinal nerve fiber layers and poorer blood flow in the retinal–bulbar vessels with clinical progression among glaucoma patients [44]. Lastly, it is important to note that certain mindfulness-based relaxation interventions, particularly those involving certain postures of the head, could potentially be counterproductive to glaucoma management and result in increased intraocular pressure [11]. The mechanisms behind this phenomenon remain uncertain, as it is unclear whether it was an isolated occurrence, as described by Zaher et al., or if it resulted from the physical alignment of the head, leading to fluid accumulation within the eye. This posture-related fluid accumulation, causing increased intraocular pressure, may be too substantial to be mitigated by any reductions in intraocular pressure associated with mindfulness-based relaxation interventions, such as a decline in stress hormone levels, as previously mentioned. Nevertheless, comprehensive data in this area are limited, as our study was designed to evaluate interventions that have shown clinical effectiveness in treating patients with glaucoma. Future research should aim to explore such counteractive interventions more thoroughly, as it holds significance for individuals with glaucoma.

## 5. Conclusions

Our findings demonstrated a significant correlation between mindfulness-based relaxation interventions and IOP reduction. Our study suggests that relaxation exercises could be a promising complementary therapy for the glaucoma and OHT clinical cohort.

## Figures and Tables

**Table 1:** Characteristics of included studies.

Author	Year	Country	Study Design	Population	Mean Age (Years)	Patients	Baseline IOP	Follow-Up	Regimens
Kaluza et al. [21]	1995	Germany	RCT	POAG	52 *	23	18.1 (2.5)	6 months	16 weekly 90 min combination sessions of autogenic relaxation exercise, ocular relaxation, and visual imagination of aqueous humor drainage
Kaluza et al. [31]	1996	Germany	RCT	POAG	52 *	23	18.04 (2.51)	8 weeks	8 weekly 90 min sessions of autogenic relaxation exercise
Dada et al. [25]	2018	India	RCT	POAG	57.88 (8.17)	45	18.8 (2.34)	3 weeks	Daily 1 h MBSR for 3 weeks
Gagrani et al. [27]	2018	India	RCT	POAG	58.4 (9.9)	30	15.9 (1.8)	6 weeks	Daily 45 min MBSR for 6 weeks
Gupta et al. [28]	2019	India	RCT	Healthy population	21.07 (1.75)	15	16.93 (2.25)	6 weeks	Daily 30 min yoga ocular exercise performed 5 days a week over 6 weeks
Dada et al. [22]	2021a	India	RCT	POAG	55.97 (8.98)	30	20.16 (3.3)	3 weeks	Daily 45 min MBSR for 3 weeks
Dada et al. [24]	2021b	India	RCT	POAG	53.23 (8.4)	30	18.06 (1.38)	6 weeks	Daily 45 min MBSR for 6 weeks
Udenia et al. [33]	2021	India	RCT	POAG	57.92 (10.18)	45	20.85 (3.39)	1, 3, 6 months	Daily 30 min YPDB exercises for up to 6 months (28 weeks)
Dada et al. [26]	2022	India	RCT	OHT	52.37 (5.99)	30	23.05 (1.17)	6 weeks	Daily 1 h MBSR for 6 weeks
Ismail et al. [29]	2022	Egypt	RCT	POAG, OHT	57.83 (3.06)	30	26.06 (2.16)	1 month	Daily 55 min Jyoti-Trataka yogic ocular exercise for 1 month
Sankalp et al. [32]	2022	India	RCT	POAG	45.29 (15.67)	34	16.26 (2.98)	14 days, 28 days	Daily 60 min Jyoti-Trataka yogic ocular exercise for 28 days
Ismail et al. [30]	2023	Egypt	RCT	POAG, OHT	69.50 (4.08)	30	25.7 (2.33)	4 weeks	Twice daily 30 min alternate-nostril breathing exercise for 4 weeks
Dada et al. [23]	2024	India	RCT	POAG	52.3 (8.2)	40	18.09 (2.8)	6 weeks	Three times daily 5 min “365 deep breathing” exercise for 6 weeks

RCT, randomized controlled trial; MBSR, mindfulness-based stress reduction; POAG, primary open-angle glaucoma; OHT, ocular hypertension; IOP, intraocular pressure. * In instances where the mean age and standard deviation were not available, the median age was utilized as an alternative measure.

## Data Availability

All data analyzed in this review are cited in the article. The protocol was preregistered on PROSPERO (CRD42024520372).

## Supplementary Materials

The following supporting information can be downloaded at: https://www.mdpi.com/article/10.3390/jcm13092591/s1, Supplementary Figure S1. Proposed pathophysiology between mental health conditions and glaucoma. Supplementary Figure S2. Overview of study design. Supplementary Figure S3. PRISMA flow diagram of the literature search and study selection. Supplementary Figure S4. Risk of bias summary for each study based on the Cochrane bias assessment tool. Supplementary Figure S5. Forest plot presenting subgroup analysis based on the baseline IOPs. Supplementary Figure S6. Forest plot presenting subgroup analysis based on the follow-up time points. Supplementary Figure S7. Funnel plot of included studies based on Hedges’ g before and after relaxation exercise.
