# Supplementary material for: The Clinical Efficacy of Different Relaxation Exercises on Intraocular Pressure Reduction: A Meta-Analysis
Source: J Clin Med. 2024 Apr 28;13(9):2591. doi: 10.3390/jcm13092591 (PMC11084912; doi:10.3390/jcm13092591)
Supplement: Supplementary file 1 [file jcm-13-02591-s001.zip › jcm-2959813-supplementary.pdf]

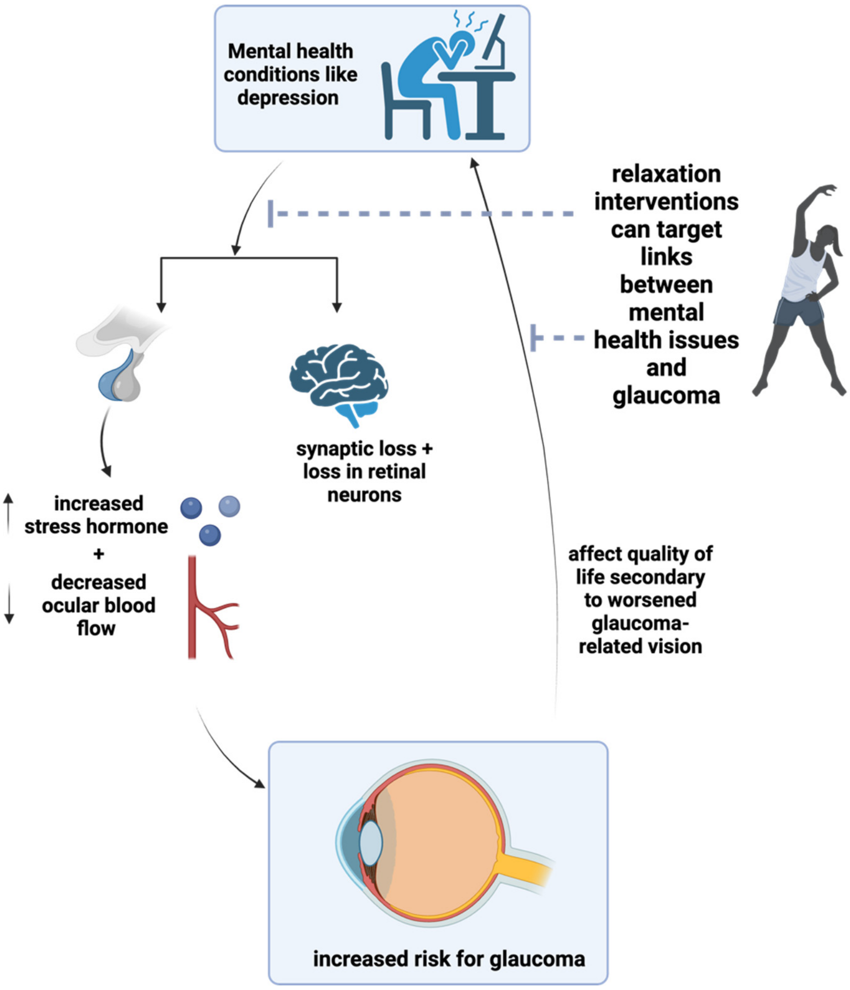

Supplementary Figure S1. Proposed pathophysiology between mental health conditions and glaucoma.

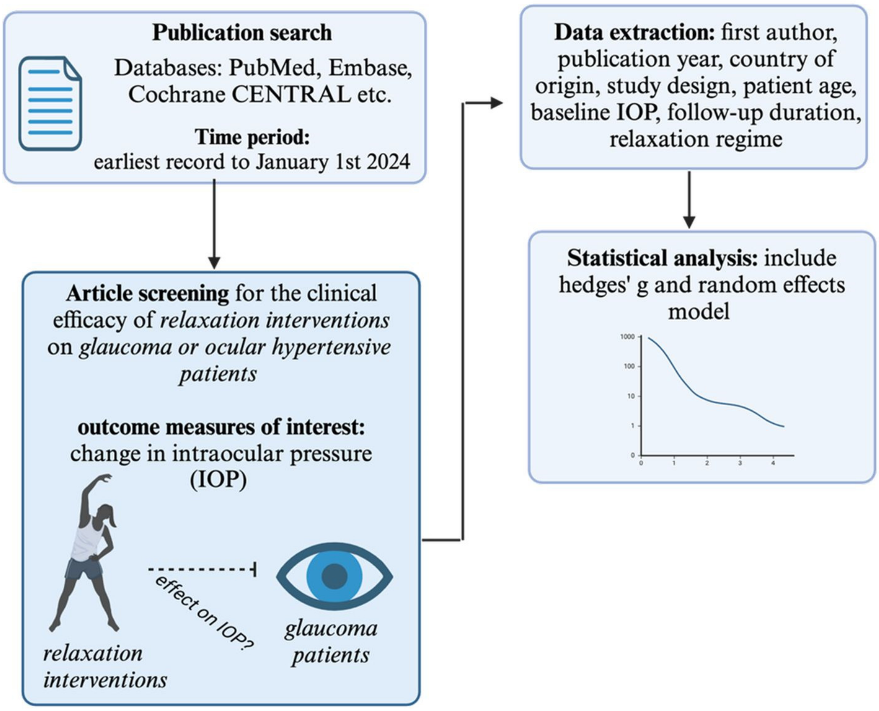

Supplementary Figure S2. Overview of study design**.**

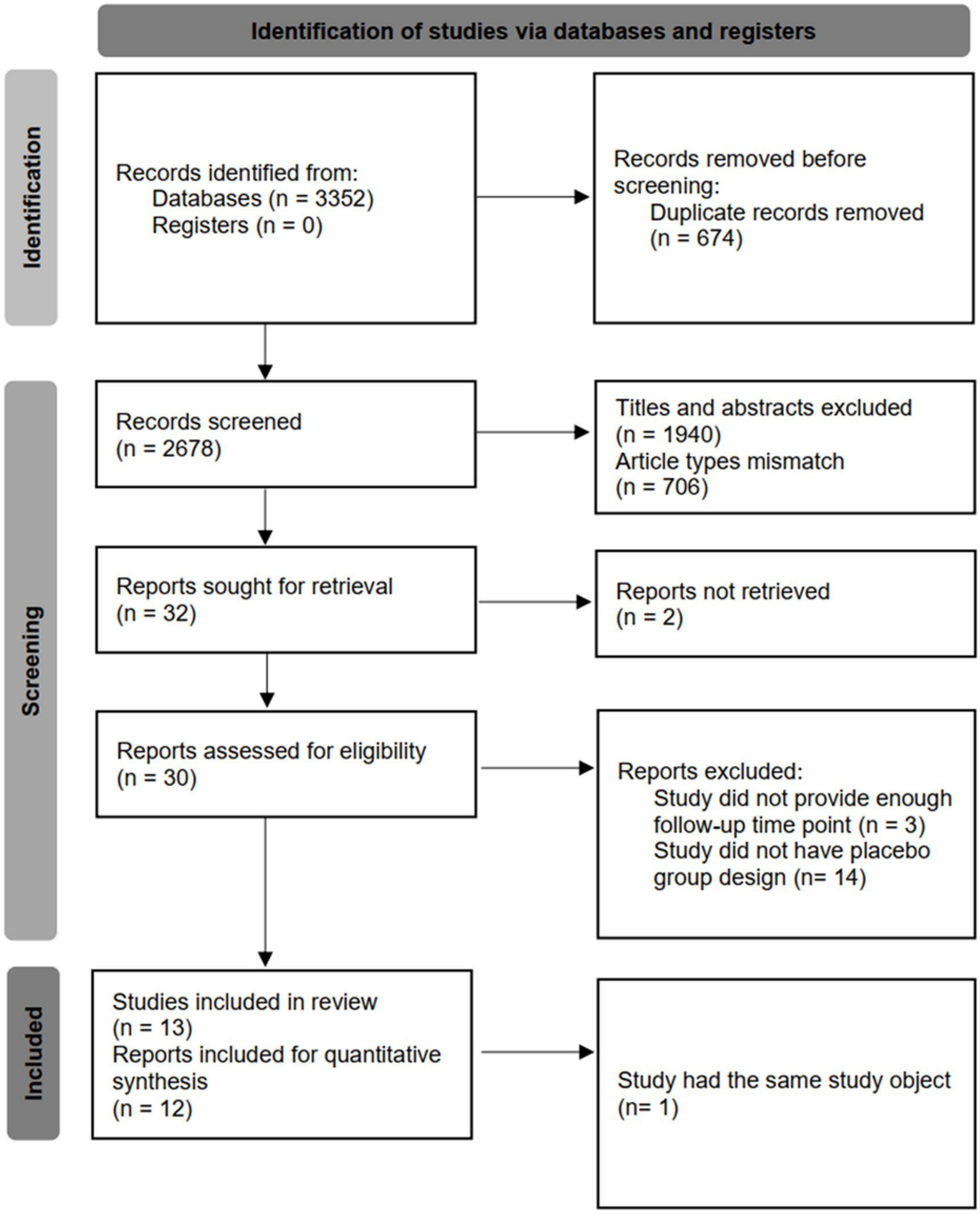

Supplementary Figure S3. PRISMA flow diagram literature search and study selection.

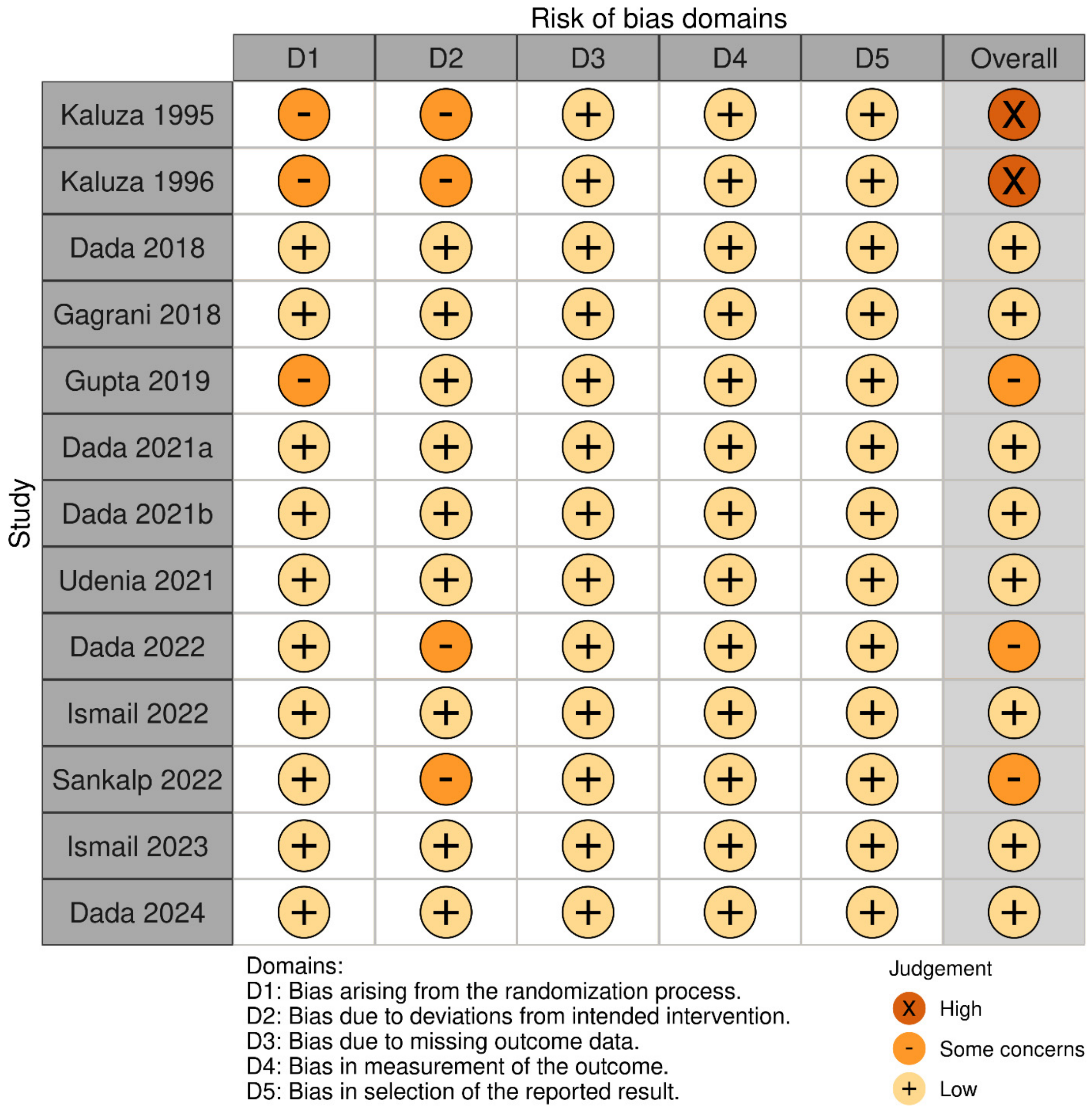
Risk of bias domains
D1
D2
D3
D4
D5
Overall
Study
Kaluza 1995
Kaluza 1996
Dada 2018
Gagrani 2018
Gupta 2019
Dada 2021a
Dada 2021b
Udenia 2021
Dada 2022
Ismail 2022
Sankalp 2022
Ismail 2023
Dada 2024
Domains:
D1: Bias arising from the randomization process.
D2: Bias due to deviations from intended intervention.
D3: Bias due to missing outcome data.
D4: Bias in measurement of the outcome.
D5: Bias in selection of the reported result.
Judgement
High
Some concerns
Low

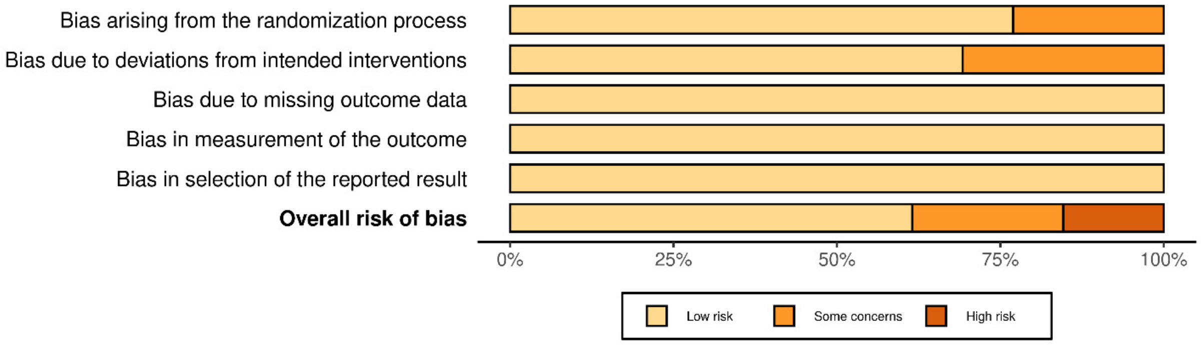
Bias arising from the randomization process
Bias due to deviations from intended interventions
Bias due to missing outcome data
Bias in measurement of the outcome
Bias in selection of the reported result
Overall risk of bias
0%
25%
50%
75%
100%
Low risk
Some concerns
High risk

Supplementary Figure S4. Risk of bias summary for each study based on the Cochrane bias assessment tool.

Group by whether baseline IOP>21 or not?	Year, study name	Hedges's g	Variance	Lower limit	Upper limit
no	1995, Kaluza et al.	-1.390	0.105	-2.025	-0.755
no	2018, Dada et al.	-2.197	0.070	-2.717	-1.676
no	2018, Gagrani et al.	-0.965	0.073	-1.494	-0.437
no	2019, Gupta et al.	-1.617	0.170	-2.424	-0.809
no	2021a, Dada et al.	-1.725	0.090	-2.313	-1.138
no	2021b, Dada et al.	-1.257	0.078	-1.805	-0.709
no	2021, Udenia et al.	-1.108	0.051	-1.548	-0.667
no	2022, Sankalp et al.	-0.119	0.058	-0.589	0.351
no	2024, Dada et al.	-0.781	0.053	-1.232	-0.331
no		-1.220	0.045	-1.633	-0.806
yes	2022, Dada et al.	-2.922	0.136	-3.645	-2.199
yes	2022, Ismail et al.	-0.943	0.072	-1.470	-0.416
yes	2023, Ismail et al.	-0.630	0.068	-1.142	-0.118
yes		-1.473	0.399	-2.711	-0.234

(Statistics for each study)

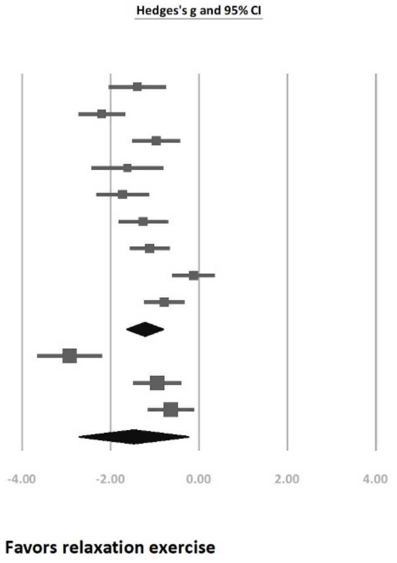

Supplementary Figure S5. Forest plot presenting subgroup analysis based on the baseline IOPs. The baseline IOPs were divided into over or less than 21 mmHg.

Group by over 1 month	Year, study name	Hedges's g	Variance	Lower limit	Upper limit
no	2018, Dada et al.	-2.197	0.070	-2.717	-1.676
no	2021a, Dada et al.	-1.725	0.090	-2.313	-1.138
no	2022, Sankalp et al.	-0.119	0.058	-0.589	0.351
no	2023, Ismail et al.	-0.630	0.068	-1.142	-0.118
no		-1.161	0.242	-2.126	-0.196
yes	1995, Kaluza et al.	-1.390	0.105	-2.025	-0.755
yes	2018, Gagrani et al.	-0.965	0.073	-1.494	-0.437
yes	2019, Gupta et al.	-1.617	0.170	-2.424	-0.809
yes	2021b, Dada et al.	-1.257	0.078	-1.805	-0.709
yes	2021, Udenia et al.	-1.108	0.051	-1.548	-0.667
yes	2022, Dada et al.	-2.922	0.136	-3.645	-2.199
yes	2022, Ismail et al.	-0.943	0.072	-1.470	-0.416
yes	2024, Dada et al.	-0.781	0.053	-1.232	-0.331
yes		-1.324	0.042	-1.723	-0.924

(Statistics for each study)

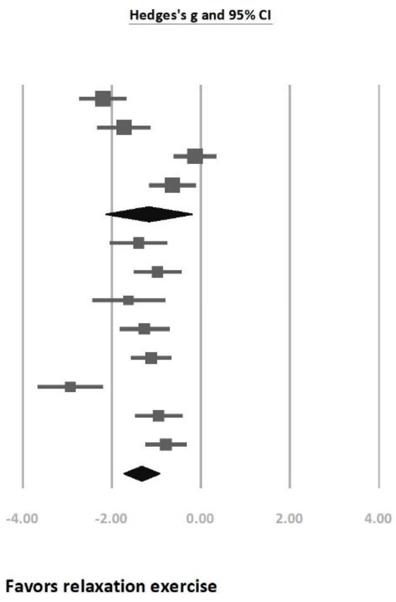

Supplementary Figure S6. Forest plot presenting subgroup analysis based on the follow-up time points. The follow-up time points were divided into over or less than one-month interval.

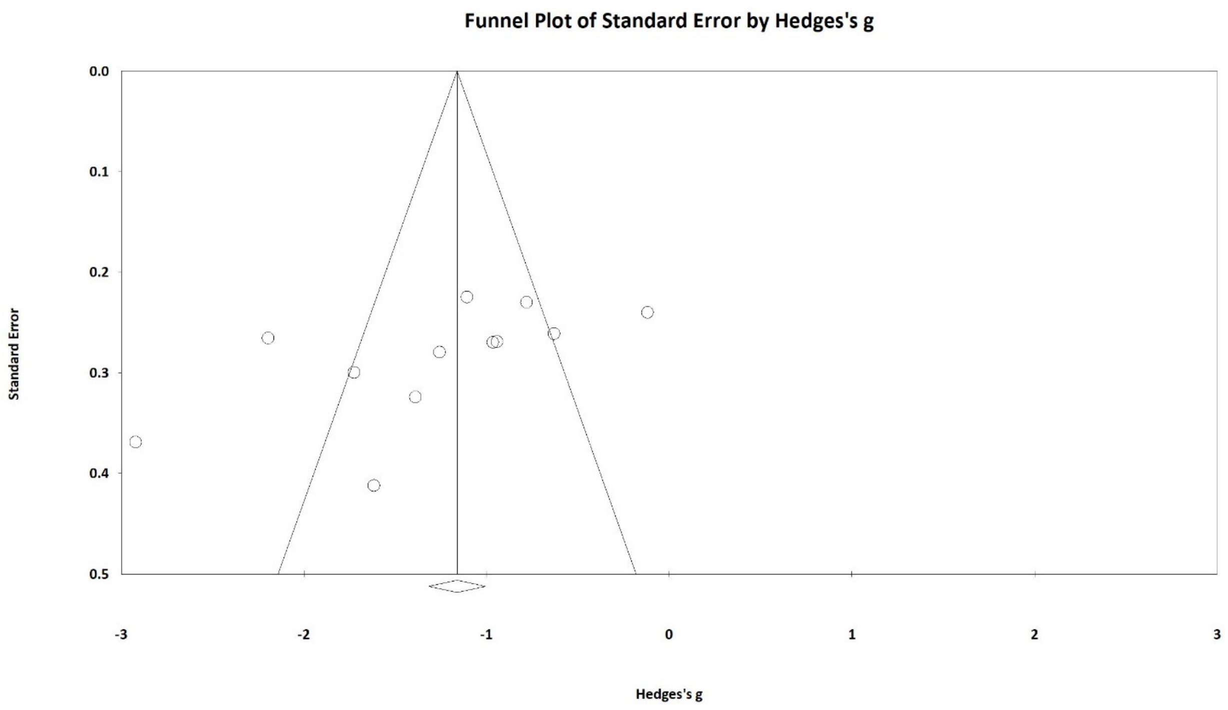

Supplementary Figure S7. Funnel plot of included studies based on Hedges' g before and after relaxation exercise.
